# Author Correction: WAVY GROWTH Arabidopsis E3 ubiquitin ligases affect apical PIN sorting decisions

**DOI:** 10.1038/s41467-022-33198-9

**Published:** 2022-09-16

**Authors:** Nataliia Konstantinova, Lukas Hoermayer, Matouš Glanc, Rabab Keshkeih, Shutang Tan, Martin Di Donato, Katarzyna Retzer, Jeanette Moulinier-Anzola, Max Schwihla, Barbara Korbei, Markus Geisler, Jiří Friml, Christian Luschnig

**Affiliations:** 1 Department of Applied Genetics and Cell Biology, Institute of Molecular Plant Biology, University of Natural Resources and Life Sciences, Vienna (BOKU), Muthgasse 18, 1190 Wien, Austria; 2 Department of Plant Biotechnology and Bioinformatics, Ghent University, 9052 Ghent, Belgium; 3 VIB Center for Plant Systems Biology, 9052 Ghent, Belgium; 4 Institute of Science and Technology Austria (IST Austria), 3400 Klosterneuburg, Austria; 5 Department of Biology, University of Fribourg, Chemin du Musée 10, 1700 Fribourg, Switzerland; 6 Present Address: School of Life Sciences, Division of Life Sciences and Medicine, University of Science and Technology of China, Hefei, 230026 China; 7 Present Address: Institute of Experimental Botany of the Czech Academy of Sciences, Rozvojová 263, Praha 6, Czech Republic

**Keywords:** Plant polarity, Tropism, Plant cell biology

Correction to: *Nature Communications* 10.1038/s41467-022-32888-8, published online 01 September 2022

In this article the author name Lukas Hoermayer was incorrectly written as Lukas Hörmayer. The original article has been corrected.

