# Supplementary material for: WAVY GROWTH Arabidopsis E3 ubiquitin ligases affect apical PIN sorting decisions
Source: Nat Commun. 2022 Sep 1;13:5147. doi: 10.1038/s41467-022-32888-8 (PMC9437102; doi:10.1038/s41467-022-32888-8)
Supplement: Supplementary file 1 — Supplementary Information [file 41467_2022_32888_MOESM1_ESM.pdf]

Supplementary Information for:

'WAVY GROWTH Arabidopsis E3 ubiquitin ligases affect apical PIN sorting decisions'

Konstantinova et al.

SUPPLEMENTARY TABLES

Supplementary Table 1: Segregation of cotyledon and seedling growth arrest phenotypes

**genotype**	**∑ individuals**	**mono/tricots, fused cotyledons**	**no cotyledons**	**seedling growth arrest**
wild type	72	0	0	0
pid-14/+	105	21	0	1
wav triple	69	0	0	0
pid-14/+ wag2-1	84	14	2	1
pid-14/+ wag1-1 wag2-1	84	0	23	1
wav triple pid-14/+	91	1	27	6
wav triple pid-14/+ wag2-1	107	0	29	16

Supplementary Table 2: Oligonucleotides used for genotyping and DNA cloning

oligo ID	sequence	application
wav3-1F	5'-ATTCTGAGATGCAGAGTGAT-3'	genotyping
wav3-1R	5'-GTTGCTTAATATGTTTCTTACTCTG-3'	genotyping
wavh1-1F	5'-ACAACAACACACGAGTCACG-3'	genotyping
wavh1-1R	5'-CGGGAGATATCGGAGATGAA-3'	genotyping
wavh2-1F	5'-CTCACAGGAAATGGTGTTTG-3'	genotyping
wavh2-1R	5'-GTCGTTGTACACTCTCAGTG-3'	genotyping
pid-14F	5'-CTTGCATCGTTATGGAATATT-3'	genotyping
pid-14R	5'-CCGTCGGATCTAACTAAGATA-3'	genotyping
wag1-1F	5'-ATGGAAGACGACGGTTATTACC-3'	genotyping
wag1-1R	5'-GGAGAAACAACCGCCACCACG-3'	genotyping
wag1-1F	5'-AACGACAGACTCTCAACCGCAA-3'	genotyping
wag2-1F	5'-GACACCGATCTTGATCTCAGCT-3'	genotyping
wag2-1R	5'-GGAATGTAAGTCTCCATTGGG-3'	genotyping
eir1-4F	5'-GGAAGCCAACGCGAAGAAT-3'	genotyping
eir1-4R	5'-GTGACATTATTATTAGTACTTACTTGA-3'	genotyping
LB	5'-TGGTTCACGTAGTGGGCCATCG-3'	genotyping
WAVH1Xmaf	5'-GGCCCGGGATGTTAAACGGCTGGAGAAGAG-3'	*WAVH1* cds cloning
WAVH1Xmar	5'-GCCCCCGGGTAAATCTAGCGTTTTCGAAACCATG-3'	*WAVH1* cds cloning
WAVH1pf	5'-GCGAATTCAAGTTAAAATTGGCTCACAT-3'	*WAHV1* promoter cloning
WAVH1pr	5'-GCGAATTCTTCTTCTTCTTCTTCAACCTCGTG-3'	*WAHV1* promoter cloning
WAV3AGT-f	5'-GGGGTACCAATGGGTACTGGTTGGCGACG-3'	*WAV3* promoter fragment cloning
WAV3-Kpn-r	5'-GCGGTACCTTAAAATCTAGCGTTTTCG-3'	*WAV3* promoter fragment cloning
W3SacIIr	5'-TGCTTGTCGAGATCCGGCGCCGCGGCAT-3'	*WAV3* promoter fragment cloning
W3pSacIfno4	5'-GTTGAGCTCGGCTGTTGAATTCTCTATTGCGG-3'	*WAV3* promoter fragment cloning
WAVSpe-f	5'-TGGCGACGAGCCTTTTGCACCACCGCCACTAGTAACAGCGATGCCGCGG-3'	*WAV3* cds cloning
WAVSpe-r	5'-CCGCGGCATCGCTGTTACTAGTGGCGGTGGTGCAAAAGGCTCGTCGCCA-3'	*WAV3* cds cloning
attB1WAV3-f	5'-GGGGACAAGTTTGTACAAAAAAGCAGGCTTTATGGGTACTGGTTGGCGACGAG-3'	*WAV3* cDNA Gateway cloning
attB2WAV3-r	5'-GGGGACCACTTTGTACAAGAAAGCTGGGTTTTAAAATCTAGCGTTTTCGAAGC-3'	*WAV3* cDNA Gateway cloning
VenSpe-f	5'-GGACTAGTGTGAGCAAGGGCGAGGAGCTG-3'	Venus for N-terminal *WAV3* fusion
VenSpe-r	5'-GGACTAGTGTACAGCTCGTCCATG-3'	Venus for N-terminal *WAV3* fusion
WAVH2f2	5'-GGCCGGGCATGCACGCACGAAGTCATGT-3'	genomic *WAVH2* fragment cloning
WAVH2r1	5'-GGCCCGGGTAAATCTGGCGTTTTCCAAGCCGTG-3'	genomic *WAVH2* fragment cloning
SOR1GWf2	5'-GGGGACAAGTTTGTACAAAAAAGCAGGCTTTATGGGGACGGGGTGGCGGAGGGCA-3'	cloning of SOR1-GFP fusion
SOR1GWR2	5'-GGGGACCACTTTGTACAAGAAAGCTGGGTTTCAGAATCTGGCGTTCTCGAAACCGTGG-3'	cloning of SOR1-GFP fusion
OPL2F	5′-GGGGACAAGTTTGTACAAAAAAAGCAGGCTTGATGGTTATGAATAATCCTGC-3'	cloning of OPL2-YFP fusion
OPL2R	5'-GGGGACCACTTTGTACAAGAAAGCTGGGTTGTAAAAGTTCATAACATTTC-3'	cloning of OPL2-YFP fusion
PIDqf-1	5'-TCCTTTCTCTCAAACCTCACC-3'	*PID* qPCR
PIDqr-1	5'-CGTTTTCTCCATCTCTGCTC-3'	*PID* qPCR
PIDqf-2	5'-CTTCCTTTCTCTCAAACCTCAC-3'	*PID* qPCR
PIDqr-2	5'-CTTTATCCACAACTTTCATCGC-3'	*PID* qPCR
Actin2/8-F	5'-ACGGTAACATTGTGCTCAGTGGTG-3'	qPCR
Actin2/8-R	5'-CTTGGAGATCCACATCTGCTGGA-3'	qPCR
EF1a-f	5'-TGAGCACGCTCTTCTTGCTTTCA-3'	qPCR
EF1a-r	5'-GGTGGTGGCATCCATCTTGTTACA-3'	qPCR
pP2-ageF	5'-GCGCACCGGTATCTTTCAATAGTTTCATCC-3'	*OPL2* cloning
pP2-xhoR	5'-CGCGCTCGAGTTTTGATTTACTTTTTCCGG-3'	*OPL2* cloning
OPL2_B1-F	5'-GGGGACAAGTTTGTACAAAAAAGCAGGCTTGATGGTTATGAATAATCCTGC-3'	*OPL2* cloning
OPL2_B2-R	5'-GGGGACCACTTTGTACAAGAAAGCTGGGTTGTAAAAGTTCATAACATTTC-3'	*OPL2* cloning

SUPPLEMENTARY FIGURES

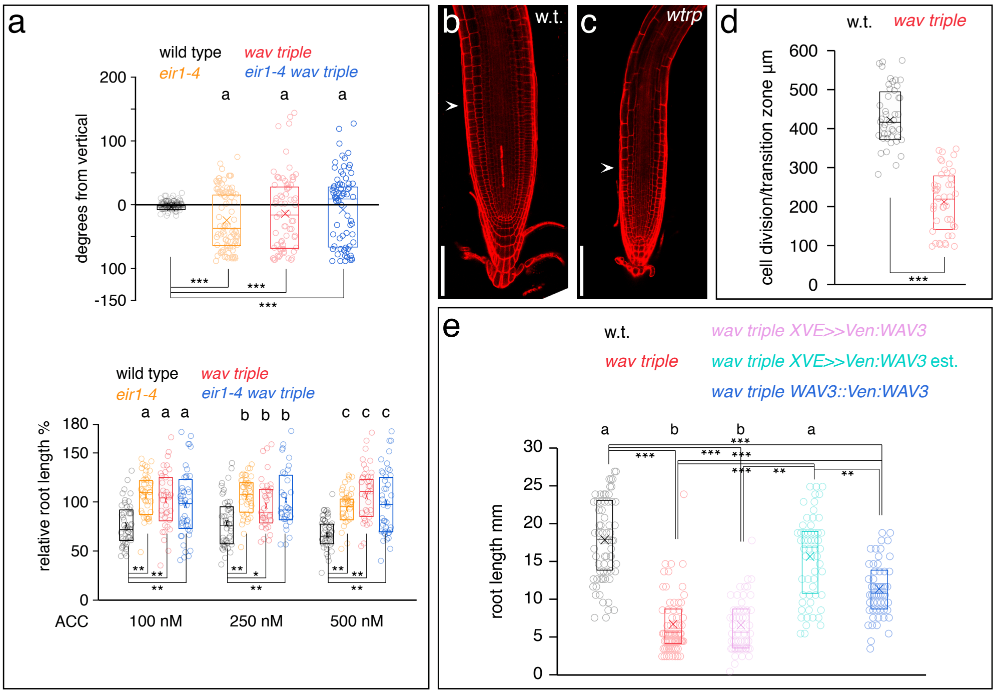

Supplementary Figure 1. Characterization of *wav triple*. **a)** Orientation of root tips, expressed as degrees deviation from vertical of 5 DAG wild type (n = 87), *eir1-4* (n = 86), *wav triple* (n = 71) and *eir1-4 wav triple* (n = 73) seedlings (top; from 3 experiments). Primary root elongation of 7 DAG wild type (100 nM ACC, n = 49; 250 nM ACC, n = 49; 500 nM ACC, n = 48), *eir1-4* (100 nM ACC, n = 42; 250 nM ACC, n = 43; 500 nM ACC, n = 39), *wav triple* (100 nM ACC, n = 36; 250 nM ACC, n = 34; 500 nM ACC, n = 35) and *eir1-4 wav triple* (100 nM ACC, n = 45; 250 nM ACC, n = 32; 500 nM ACC, n = 37) seedlings in presence of ACC (bottom; root length on control = 100%). **b,c)** Propidium iodide staining of wild type (w.t.) and *wav triple* (*wtrp*) primary root meristem at 5 DAG. Arrowheads indicate onset of cell elongation. Comparable results were obtained in 5 experimental repeats **d)** Comparison of root meristem cell division/transition zone size (defined as the area with essentially isodiametric shape of epidermis cells) of wild type (w.t.; n = 44) and *wav triple* (*wtrp*; n = 43) seedlings at 5 DAG. **e)** Primary root length of 5 DAG wild type (w.t.; n = 67), *wav triple* (n = 69), *wav triple XVE>>Ven:WAV3* on control medium (n = 55) and in presence of 2 µM estradiol ('est.'; n = 58), and of *wav triple WAV3::Ven:WAV3* seedlings (n = 56). Circles represent single data points; boxes: first and third quartiles; center line: median; 'x': mean value. Two-tailed t-test was performed to determine the significance of differences between data sets (d). One-way ANOVA tests were employed to determine the difference between the averages (e); Levene's tests were employed to determine the equality of variances (a); **: $p < 0.01$; ***: $p < 0.001$; a,b,c: $p > 0.05$. Size bars: b,c = 50 µm. Source data are provided as Source Data file.

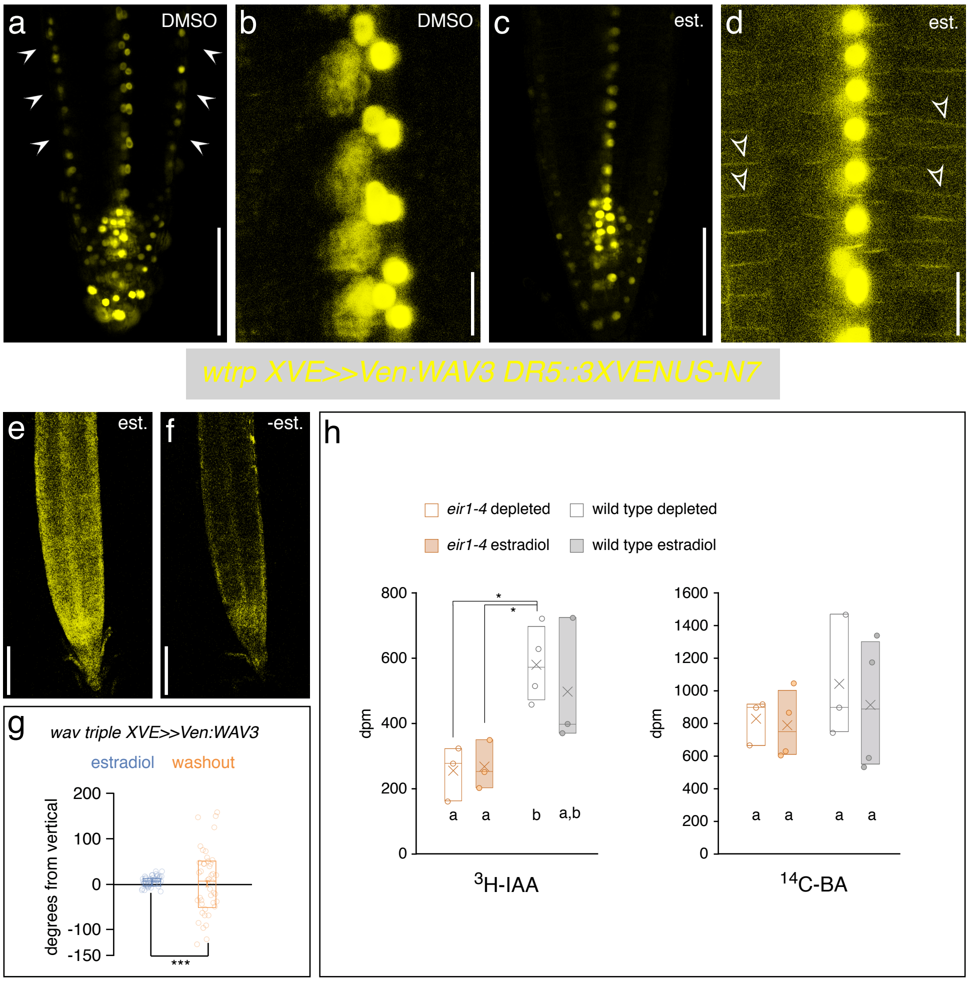

Supplementary Figure 2. Auxin distribution in *wav triple*. **a-d)** Expression of *DR5rev:3XVENUS-N7* in *wav triple XVE::Venus:WAV3* root meristems at 5 DAG. On DMSO control plates laterally located nuclear DR5-Venus signals are indicated by arrowheads (a), whereas no *XVE::Venus:WAV3* expression is visible (b). Upon incubation on 2 µM estradiol ('est.'; c,d) Venus:WAV3 expression is clearly visible (d, open arrowheads). Similar results were observed in 3 experimental repeats on DMSO and on estradiol. **e,f)** Reporter gene expression in *wav triple XVE>>Ven:WAV3* root meristems grown on 2 µM estradiol ('est.') for 5 days, followed by washout in liquid growth medium and transfer onto fresh estradiol plate (e) or on a control plate containing solvent only ('-est.'; f). Images were taken after 6 hours incubation. A reproducible downregulation of Venus reporter signals was detected in 5 experimental repeats. **g)** Reorientation of root growth of *wav triple XVE>>Ven:WAV3* grown on 2 µM estradiol for 5 days followed by washout and incubation on control medium (n = 43) or in presence of 2 µM estradiol (n = 47) and gravistimulated for 12 hours. Direction of root tips is expressed as degrees deviation from vertical. **h)** Root shootward (basipetal) PAT measurement using ^{3}H-IAA and as a control, ^{14}C-benzoic acid (BA), on 5 DAG wild type (BA: estradiol depleted, n = 3; estradiol, n = 4. IAA: estradiol depleted, n = 3; estradiol, n = 4. Each data point represents results from 20 pooled roots) and *eir1-4* seedlings (BA: estradiol depleted, n

= 3; estradiol, n = 4. IAA: estradiol depleted, n = 3; estradiol, n = 3. Each data point represents results from 20 pooled roots). Radiotracers were applied to the root tip followed by their quantification 5 mm above the very root tip. Assays were conducted in presence of 2 μM 17β-estradiol ('estradiol') or in presence of DMSO solvent only ('depleted'); for further details see Methods. Circles represent single data points; boxes: first and third quartiles; center line: median; 'x': mean value. Levene's tests were employed to determine the equality of variances (g). One-way ANOVA tests were employed to determine the difference between the averages (h); *: $p < 0.05$; ***: $p < 0.001$; a,b: $p > 0.05$. Size bars: a,c,e,f = 50 μm; b,d = 20 μm. Source data are provided as Source Data file.

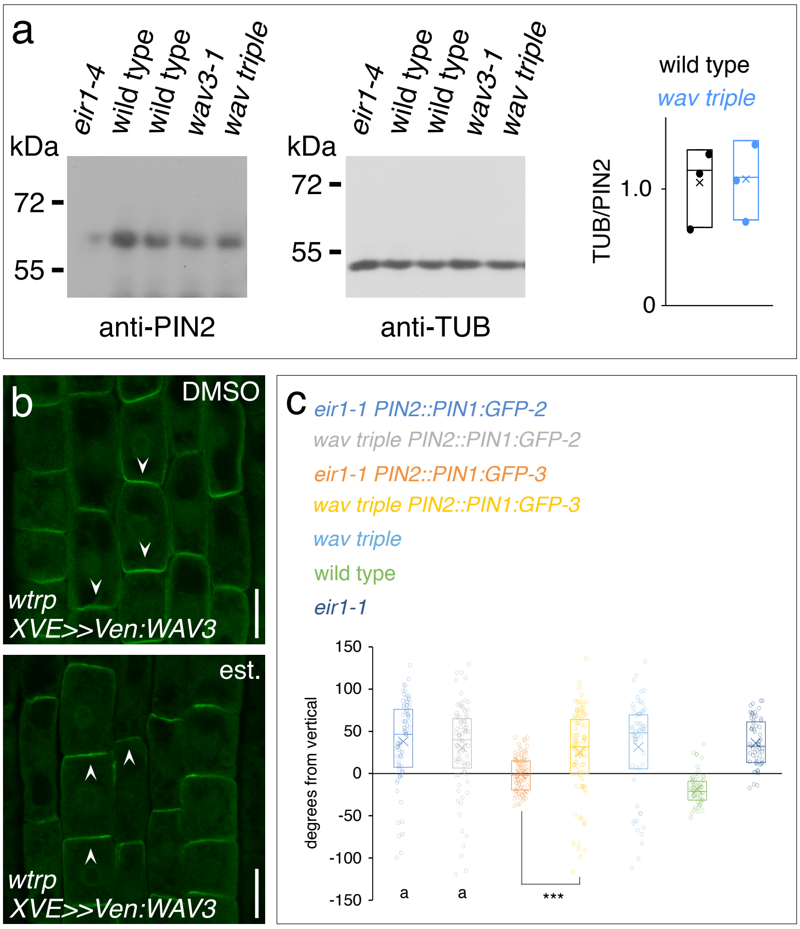

Supplementary Figure 3. PIN2 in *wav triple* and root gravitropism in *wav triple PIN2::PIN1:GFP* reporter lines. **a)** Western blots performed with 5 DAG *eir1-4*, wild type (w.t.), *wav3-1* and *wav triple* membrane protein extracts, probed with anti-PIN2. Anti-α-tubulin (TUB) was used as loading control (left panels). TUB-to-PIN2 signal ratio in 7 DAG wild type and *wav triple* root membrane protein extracts. Dots represent data from 3 biological repeats; boxes: first and third quartiles; center line: median; 'x': mean value. Two-tailed t-test was performed to test for significant differences (right panel) **b)** PIN2 immunostaining (green signals) in root meristem epidermis cells of 5 DAG *wav triple XVE>>Ven:WAV3* germinated in presence of 2 µM estradiol ('est.'; right) or solvent only ('DMSO'; left). Arrowheads indicate polarity of PIN2 signals. Similar PIN2 polarity switches were observed in 5 experimental repeats. **c)** Orientation of root growth of 5 DAG *eir1-1 PIN2::PIN1:GFP-2* (n = 80), *wav triple PIN2::PIN1:GFP-2* (n = 78), *eir1-1 PIN2::PIN1:GFP-3* (n = 87), *wav triple PIN2::PIN1:GFP-3* (n = 92), *wav triple* (n = 59), wild type (n = 59) and *eir1-1* (n = 59). Direction of root tips is expressed as degrees deviation from vertical. Circles represent single data points; boxes: first and third quartiles; center line: median; 'x': mean value. Levene's tests were employed to determine the equality of variances of *PIN2::PIN1:GFP-2* and *PIN2::PIN1:GFP-3* data sets, respectively; ***: $p < 0.001$; a: $p > 0.05$. Source data are provided as Source Data file.

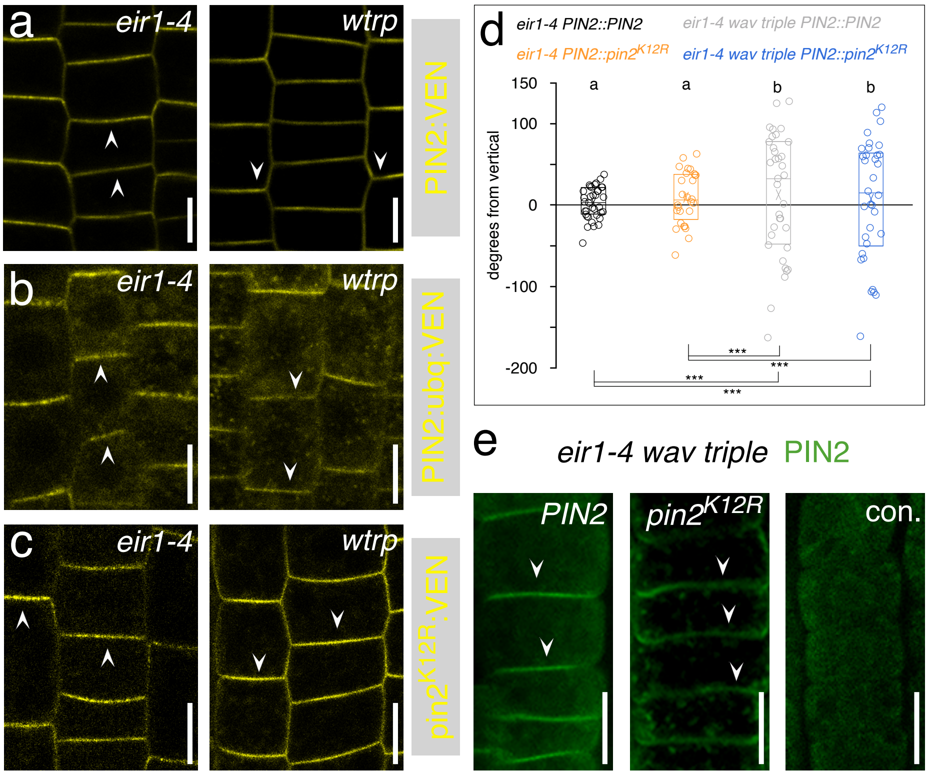

Supplementary Figure 4. Analysis of *wav triple* lines expressing *pin2* alleles with compromised ubiquitylation status. **a-c)** Root meristem cells of 5 DAG *eir1-4* and *wav triple* (*wtrp*) expressing *PIN2::PIN2:VEN* (a), *PIN2::PIN2:ubq:VEN* (b) and *PIN2::pin2^{K12R}:VEN* (c). **d)** Orientation of root growth of 5 DAG *eir1-4 PIN2::PIN2* (n = 36), *eir1-4 wav triple PIN2::PIN2* (n = 35), *eir1-4 PIN2::pin2^{K12R}* (n = 28) and *eir1-4 wav triple PIN2::pin2^{K12R}* (n = 34). Direction of root tips is expressed as degrees deviation from vertical. Similar results were obtained in 3 independent experiments. **e)** PIN2 immunolocalization in *eir1-4 wav triple* root meristem epidermis cells expressing *PIN2::PIN2* (left), *PIN2::pin2^{K12R}* (middle) and control ('con.') lacking a reporter construct, at 5 DAG. Comparable results were obtained in 3 immunostaining experiments. Arrowheads indicate polarity of reporter protein signals at the PM. Circles represent single data points; boxes: first and third quartiles; center line: median; 'x': mean value. Levene's tests were employed to determine the equality of variances; ***: $p < 0.001$; a,b: $p > 0.05$. Size bars: a-c,e = 10 µm. Source data are provided as Source Data file.

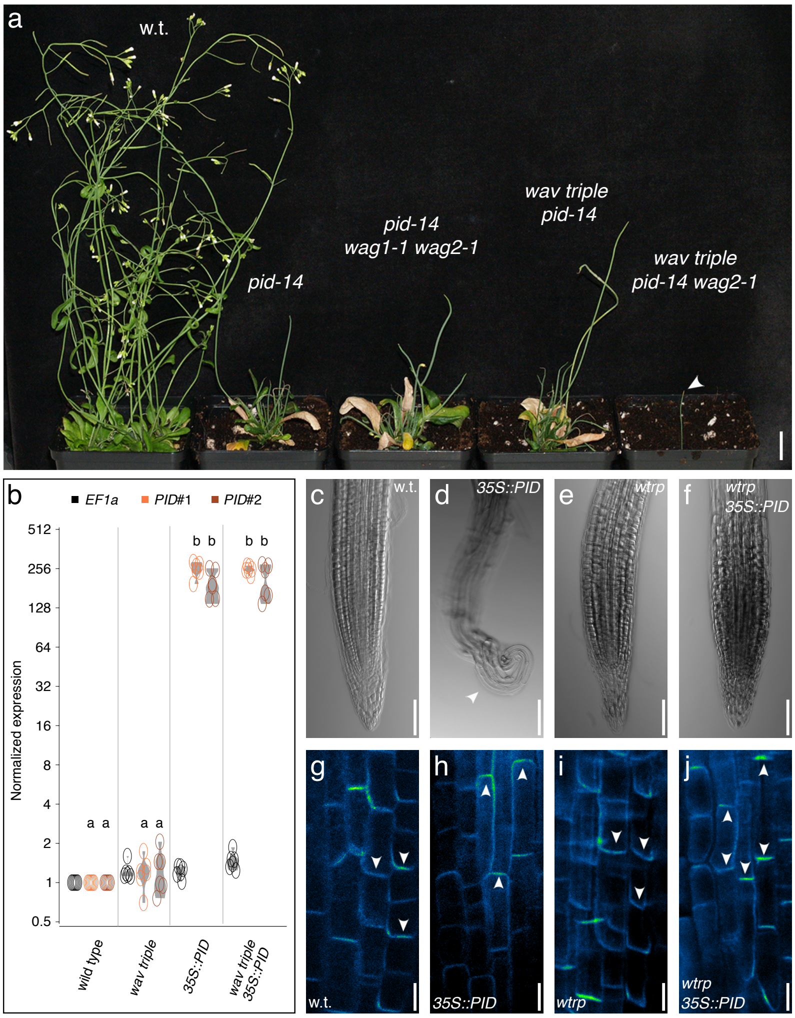

Supplementary Figure 5. Crosstalk between *WAV3/WAVH* and *PID/WAG* genes. **a)** From left-to-right: Flowering wild type (w.t.; 28 DAG), *pid-14*, *pid-14 wag1-1 wag2-1*, *wav triple pid-14*, *wav triple pid-14 wag2-1* (all 38 DAG). Arrowhead indicates pin-like inflorescence of *wav triple pid-14 wag2-1* plantlet. **b)** *PID* transcript levels in wild type, *wav triple*, *35S::PID* and *wav triple 35S::PID* seedlings at 7 DAG. Six repetitions with two different *PID* primer pairs (*PID*#1 and *PID*#2) and an *EF1a* (At1g07940) primer pair were made for each sample. Transcript levels were normalized to expression of *ACT2* (At3g18780). Wild type transcript levels were set to one; circles represent individual repetitions. One-way ANOVA tests were employed to determine the difference between the averages**;** a,b: $p > 0.05$. **c-f)** 5 DAG primary root meristems of wild type (w.t.; c), *35S::PID* (d), *wav triple* (*wtrp*; e) and *wav triple 35S::PID* (*wtrp*; f). Arrowhead indicates root meristem consumption in *35S::PID*. Comparable results were observed in 3 experimental repeats, with a total of 12 root meristems analyzed for each line. **g-j)** PIN1 immunolocalization in root meristem stele cells of 4 DAG wild type (w.t.; g), *35S::PID* (h), *wav triple* (*wtrp*; i) and *wav triple 35S::PID* (*wtrp*; j). Arrowheads indicate polarity of PIN1 localization. Comparable results were observed in 3 separate immunostaining experiments performed with all 4 lines. Size bars: a = 10 mm; c-f = 20 µm; g-j = 5 µm. Source data are provided as Source Data file.

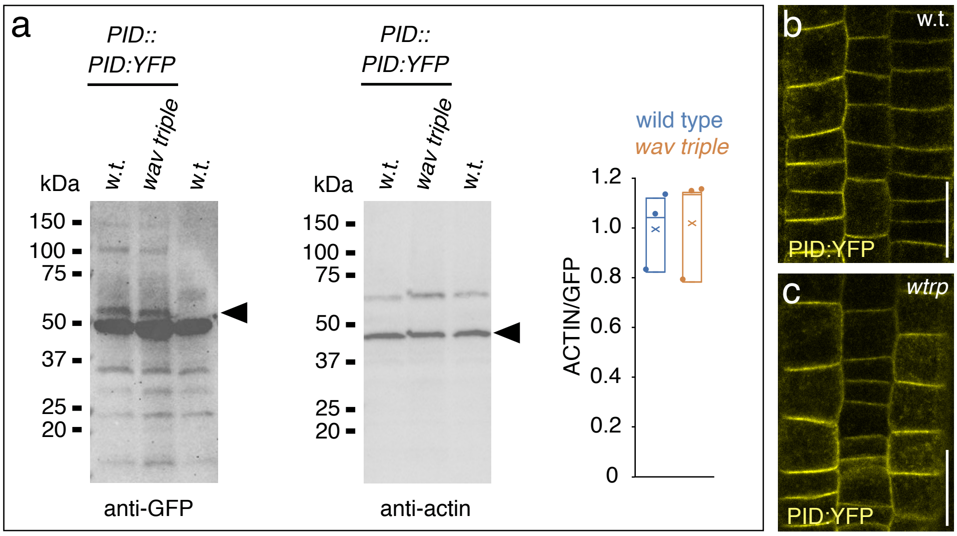

Supplementary Figure 6. *PID* expression in *wav triple*. **a)** Western blots performed with protein extracts obtained from 7 DAG wild type (w.t.), *wav triple PID::PID:YFP* and wild type *PID::PID:YFP*, probed with anti-GFP (left panel). Anti-actin (actin) was used as loading control (middle panel). Arrowheads indicate PID:YFP and actin, respectively. ACTIN-to-GFP signal ratio in 7 DAG wild type and *wav triple* root membrane protein extracts (right panel). Dots represent data from 3 biological repeats; boxes: first and third quartiles; center line: median; 'x': mean value. Two-tailed t-test was done to test for significant differences ($p > 0.05$). **b,c)** Subcellular localization of PID:YFP (yellow) in 7 DAG wild type *PID::PID:YFP* (w.t.; b) and *wav triple PID::PID:YFP* (*wtrp*; c) root meristem epidermis cells. 12 root meristems examined in 3 experiments produced similar results. Size bars: b,c = 10 µm. Source data are provided as Source Data file.

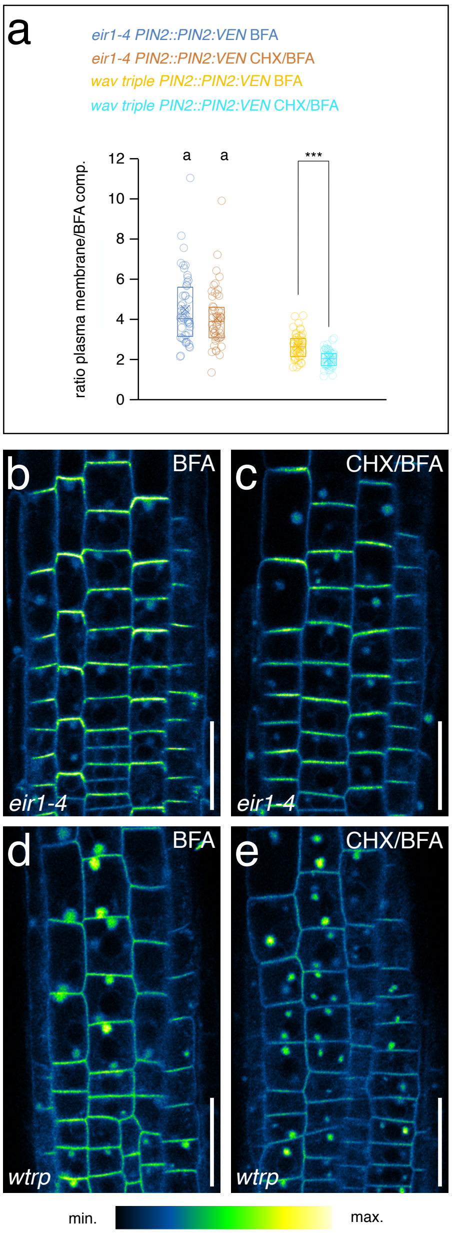

Supplementary Figure 7. Analysis of PIN2-Venus subcellular distribution upon inhibition of BFA-sensitive ARF-GEFs. **a)** Plasma membrane-to-BFA compartment signal ratio in 4 DAG *eir1-4 PIN2::PIN2:VEN* and *wav triple PIN2::PIN2:VEN* root meristem epidermis cells treated with BFA only (BFA), or pretreated with CHX followed by CHX/BFA co-treatment (CHX/BFA). For further experimental details see Methods. **b-e)** 4 DAG *eir1-4 PIN2::PIN2:VEN* (*eir1-4*; b,c) and *wav triple PIN2::PIN2:VEN* (*wtrp*; d,e) root meristem epidermis cells treated with BFA only (b,d) or pretreated with CHX followed by CHX/BFA co-treatment (c,e). Circles represent single data points; boxes: first and third quartiles; center line: median; 'x': mean value. Two-tailed t-tests were employed to test for significance; n = 51 for each line; ***: $p < 0.001$; a: $p > 0.05$. Size bars: b-e = 20 μm. Source data are provided as Source Data file.

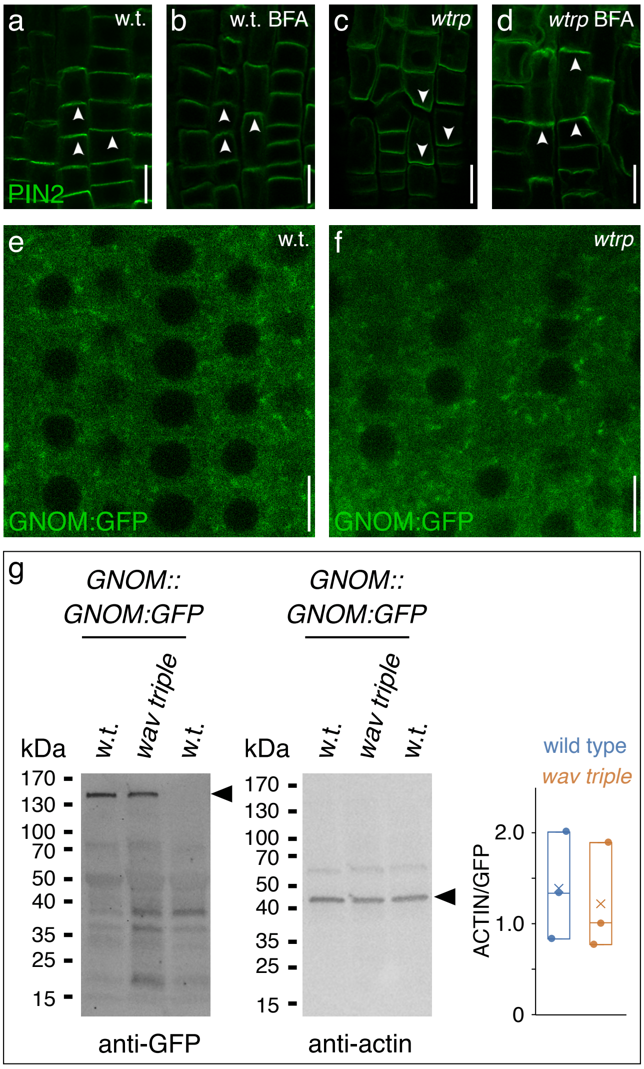

Supplementary Figure 8. Analysis of crosstalk between *wav triple* and *GNOM* **a-d)** PIN2 immuno-localization in 5 DAG wild type (w.t.; a,b) and *wav triple* (*wtrp*; c,d) root meristem epidermis cells. Seedlings were subject to treatment with 10 μM BFA for 24 hours (b,d) or incubated on control medium containing solvent only (a,c). Arrowheads indicate polarity of PIN2 signals. BFA-induced PIN2 polarity shifts were observed in 3 experimental repetitions **e,f)** Wild type (w.t.; e) and *wav triple* (*wtrp*; f) root meristem epidermis cells of 5 DAG seedlings, expressing *GNOM::GNOM:GFP*. A comparable distribution of GNOM-GFP reporter signals was observed in 3 independent experiments, with a total of 9 individuals analyzed for both genotypes. **g)** Western blots performed with protein extracts obtained from 7 DAG wild type (w.t.), *wav triple GNOM::GNOM:GFP* and wild type *GNOM::GNOM:GFP*, probed with anti-GFP (left panel). Anti-actin was used as loading control (middle panel). Arrowheads indicate GNOM:GFP and actin, respectively. ACTIN-to-GFP signal ratio in 7 DAG wild type and *wav triple* root membrane protein extracts (right panel). Dots represent data from 3 biological repeats; boxes: first and third quartiles; center line: median; 'x': mean value. Two-tailed t-test was performed to test for significance ($n = 3$; $p > 0.05$). Size bars: a-d = 20 μm; e,f = 10 μm. Source data are provided as Source Data file.

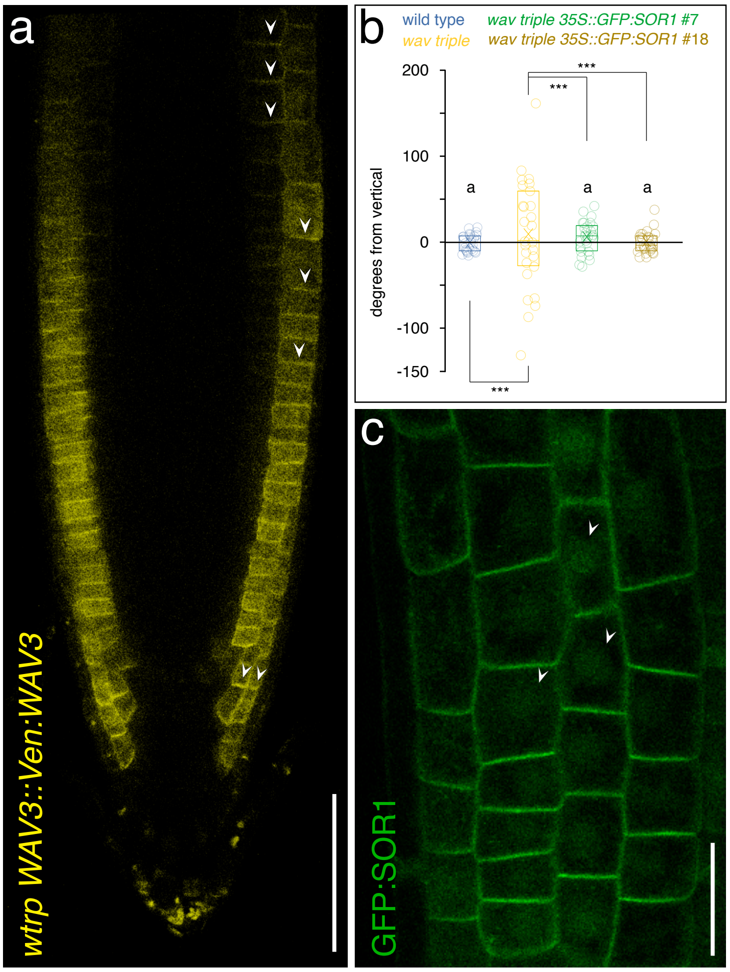

Supplementary Figure 9. Expression of *WAV3* and *SOR1* reporter genes. **a)** Subcellular Venus signal localization in a 6 DAG *wav triple* (*wtrp*) primary root meristem expressing partially complementing *WAV3::Ven:WAV3*. Arrowheads indicate basal localization in root meristem LRC, epidermis and cortex cells. A similar reporter signal distribution was observed in 3 independent experimental repeats. **b)** Orientation of root growth of 7 DAG wild type (n = 26), *wav triple* (n = 27) and two *wav triple* lines (n = 27 for each line) expressing *35S::GFP:SOR1* (#7, #18). **c)** Root meristem epidermis cells of 5 DAG *wav triple 35S::GFP:SOR1* #18. Arrowheads indicate intracellular signals of reporter protein. A reproducible GFP-SOR1 signal distribution was observed in 3 experimental repeats. Circles represent single data points; boxes: first and third quartiles; center line: median; 'x': mean value. Levene's tests were employed to determine the equality of variances; ***: $p < 0.001$; a: $p > 0.05$. Size bars: a = 50 µm; c = 20 µm. Source data are provided as Source Data file.

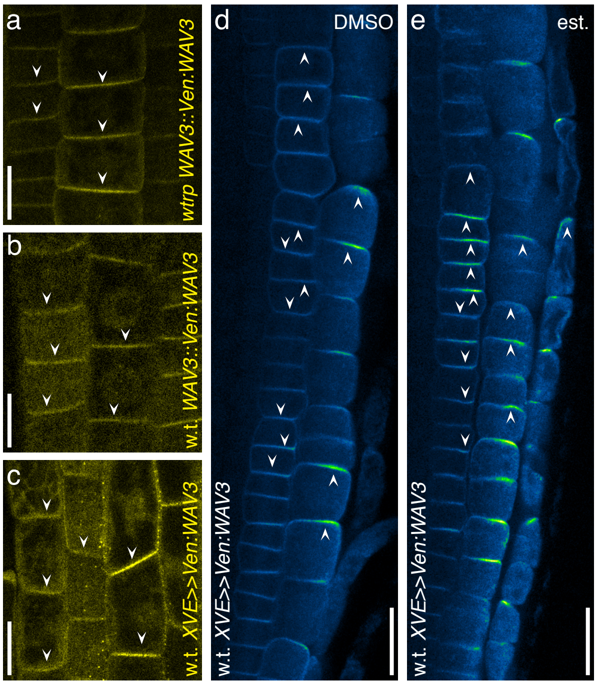

Supplementary Figure 10. Analysis of *WAV3:Venus* expression. **a,b)** Subcellular Venus signal distribution in root meristem epidermis cells of 6 DAG *wav triple* (*wtrp*; a) and wild type (w.t.; b) expressing *WAV3::Ven:WAV3*. Similar results were obtained in 3 experimental repeats, in which we compared *wav triple* and wild type expressing *WAV3::Ven:WAV3*. **c)** Subcellular Venus signal distribution in in root meristem epidermis cells of 6 DAG wild type (w.t.) expressing *XVE>>Ven:WAV3*, grown in presence of 2 μM estradiol. Comparable Venus signal distribution was observed in 3 independent experiments. **d,e**) PIN2 immunostaining in wild type (w.t.) root meristem cells expressing *XVE>>Ven:WAV3* grown on medium substituted with solvent (DMSO; d) or in presence of 2 μM estradiol (est.; e). PIN2 localization was analyzed in 3 experiments, yielding comparable results. Wild type lines expressing WAV3 reporters were obtained in the progeny of *wav triple WAV3::Ven:WAV3* or *wav triple XVE>>Ven:WAV3* crossed into Col-0. Arrowheads indicate polarity of WAV3-Venus signals (a-c) and PIN2 signals (d,e). Size bars: a-c = 10 μm; d,e = 20 μm.

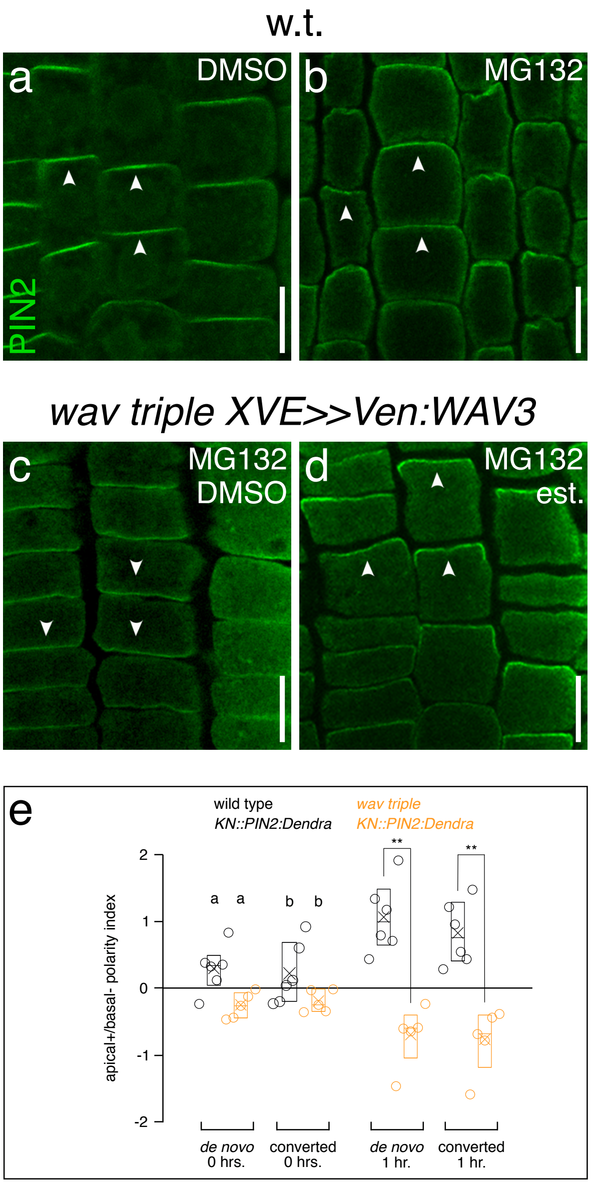

Supplementary Figure 11. Proteasome activity does not affect PIN2 polarity. **a,b)** PIN2 immunolocalization in 5 DAG root meristem epidermis cells of wild type (w.t.) incubated for 6 hours in presence of 50 μM MG132 (b) or solvent only (DMSO; a). Comparable results were obtained in 3 immunostaining experiments. **c,d)** PIN2 immunolocalization in 5 DAG root meristem epidermis cells of *wav triple XVE>>Ven:WAV3.* Seedlings germinated on 5 μM estradiol (est.; d) or solvent only (DMSO; c) were incubated in presence of 50 μM MG132 for 6 hours and then subject to immunostaining. Arrowheads indicate polarity of PIN2 signals at the PM. Comparable results were obtained in 3 immunostaining experiments. **e)** Quantification of PIN2:Dendra polarity establishment in 5 DAG *KN::PIN2:Dendra* (n = 6) and *wav triple KN::PIN2:Dendra* (n = 5) upon photoconversion (0 hours) and 1 hour after such conversion. Circles represent single data points; boxes: first and third quartiles; center line: median; 'x': mean value. One-way ANOVA with post-hoc Tukey HSD was performed (e); **: $p < 0.01$; a,b: $p > 0.05$. Size bars: a-d = 10 μm. Source data are provided as Source Data file.
