# Supplementary material for: WAVY GROWTH Arabidopsis E3 ubiquitin ligases affect apical PIN sorting decisions
Source: Nat Commun. 2022 Sep 1;13:5147. doi: 10.1038/s41467-022-32888-8 (PMC9437102; doi:10.1038/s41467-022-32888-8)
Supplement: Supplementary file 3 — Reporting Summary [file 41467_2022_32888_MOESM3_ESM.pdf]

nature portfolio

Corresponding author(s): Luschnig Christian

Last updated by author(s): 2022/08/14

Reporting Summary

Nature Portfolio wishes to improve the reproducibility of the work that we publish. This form provides structure for consistency and transparency in reporting. For further information on Nature Portfolio policies, see our Editorial Policies and the Editorial Policy Checklist.

Statistics

For all statistical analyses, confirm that the following items are present in the figure legend, table legend, main text, or Methods section.

n/a	Confirmed	
☐	☒	The exact sample size (*n*) for each experimental group/condition, given as a discrete number and unit of measurement
☐	☒	A statement on whether measurements were taken from distinct samples or whether the same sample was measured repeatedly
☐	☒	The statistical test(s) used AND whether they are one- or two-sided<br>*Only common tests should be described solely by name; describe more complex techniques in the Methods section.*
☐	☒	A description of all covariates tested
☐	☒	A description of any assumptions or corrections, such as tests of normality and adjustment for multiple comparisons
☐	☒	A full description of the statistical parameters including central tendency (e.g. means) or other basic estimates (e.g. regression coefficient) AND variation (e.g. standard deviation) or associated estimates of uncertainty (e.g. confidence intervals)
☐	☒	For null hypothesis testing, the test statistic (e.g. *F*, *t*, *r*) with confidence intervals, effect sizes, degrees of freedom and *P* value noted<br>*Give P values as exact values whenever suitable.*
☒	☐	For Bayesian analysis, information on the choice of priors and Markov chain Monte Carlo settings
☒	☐	For hierarchical and complex designs, identification of the appropriate level for tests and full reporting of outcomes
☒	☐	Estimates of effect sizes (e.g. Cohen's *d*, Pearson's *r*), indicating how they were calculated

Our web collection on statistics for biologists contains articles on many of the points above.

Software and code

Policy information about availability of computer code

Data collection	Image data was collected using Leica LAS EZ CLSM and ImageJ (NIH; http://rsb.info.nih.gov/ij) software
Data analysis	Images were analyzed using ImageJ/Fiji software (version 2.1.0/1.53c); Data was processed in MS Excel (version 16.16.4) and in Sigma Plot (Systat, Chicago, IL, USA).

For manuscripts utilizing custom algorithms or software that are central to the research but not yet described in published literature, software must be made available to editors and reviewers. We strongly encourage code deposition in a community repository (e.g. GitHub). See the Nature Portfolio guidelines for submitting code & software for further information.

Data

Policy information about availability of data

All manuscripts must include a data availability statement. This statement should provide the following information, where applicable:

- Accession codes, unique identifiers, or web links for publicly available datasets
- A description of any restrictions on data availability
- For clinical datasets or third party data, please ensure that the statement adheres to our policy

The authors declare that all data supporting the findings of this study are available within the manuscript and its Supplementary files, and are available from the corresponding author upon request.

Human research participants

Policy information about studies involving human research participants and Sex and Gender in Research.

Reporting on sex and gender	n/a
Population characteristics	n/a
Recruitment	n/a
Ethics oversight	n/a

Note that full information on the approval of the study protocol must also be provided in the manuscript.

Field-specific reporting

Please select the one below that is the best fit for your research. If you are not sure, read the appropriate sections before making your selection.

☒ Life sciences ☐ Behavioural & social sciences ☐ Ecological, evolutionary & environmental sciences

For a reference copy of the document with all sections, see nature.com/documents/nr-reporting-summary-flat.pdf

Life sciences study design

All studies must disclose on these points even when the disclosure is negative.

Sample size	No statistics has been used for predetermination of sample size. Sample size has been determined based on comparable experiments.
Data exclusions	No data was excluded, unless individuals from our samples exhibited signs of cell death (determined by microscopy).
Replication	At least 3 independent biological repeats were done for all experiments.
Randomization	For comparison of different genotypes/growth conditions, samples were grown alongside for further comparison. Randomly picked individuals from these samples were used for data acquisition.
Blinding	Blinding was not considered for this study. Reporter signal intensities and distribution were determined using image analysis, obtained from randomly picked individual seedlings.

Reporting for specific materials, systems and methods

We require information from authors about some types of materials, experimental systems and methods used in many studies. Here, indicate whether each material, system or method listed is relevant to your study. If you are not sure if a list item applies to your research, read the appropriate section before selecting a response.

Materials & experimental systems

n/a	Involved in the study	
☐	☒	Antibodies
☒	☐	Eukaryotic cell lines
☒	☐	Palaeontology and archaeology
☒	☐	Animals and other organisms
☒	☐	Clinical data
☒	☐	Dual use research of concern

Methods

n/a	Involved in the study	
☒	☐	ChIP-seq
☒	☐	Flow cytometry
☒	☐	MRI-based neuroimaging

Antibodies

Antibodies used	rabbit anti-PIN2 (Abas et al., 2006); rabbit anti-PIN1 (Benkova et al., 2003); mouse anti-tubulin (Sigma, T6074), HRP-conjugated goat-anti-rabbit IgG (Jackson, 111-036-003); HRP-conjugated goat-anti-mouse IgG (Jackson, 115-035-003); FITC-conjugated goat-anti-rabbit IgG (DAB-088166); JL-8 anti-GFP antibody (Clontech, 632381); HRP-conjugated goat-anti-mouse (GE healthcare, GE:NXA931).
Validation	Specificity of anti-PIN1 and -PIN2 antibodies was determined in negative control lines; i.e. loss-of-function mutants, deficient in

Validation

expression of PIN1 and PIN2, respectively. For all commercially obtained antibodies used for this study, we followed the manufacturers' guidelines and recommendations.
